# The Use of Coded PCR Primers Enables High-Throughput Sequencing of Multiple Homolog Amplification Products by 454 Parallel Sequencing

**DOI:** 10.1371/journal.pone.0000197

**Published:** 2007-02-14

**Authors:** Jonas Binladen, M. Thomas P. Gilbert, Jonathan P. Bollback, Frank Panitz, Christian Bendixen, Rasmus Nielsen, Eske Willerslev

**Affiliations:** 1 Center for Ancient Genetics, Institute of Biology, University of Copenhagen, Copenhagen, Denmark; 2 Center for Bioinformatics and Institute of Biology, University of Copenhagen, Copenhagen, Denmark; 3 Department of Genetics and Biotechnology, Danish Institute of Agricultural Sciences Research Centre Foulum, Tjele, Denmark; Indiana University, United States of America

## Abstract

**Background:**

The invention of the Genome Sequence 20™ DNA Sequencing System (454 parallel sequencing platform) has enabled the rapid and high-volume production of sequence data. Until now, however, individual emulsion PCR (emPCR) reactions and subsequent sequencing runs have been unable to combine template DNA from multiple individuals, as homologous sequences cannot be subsequently assigned to their original sources.

**Methodology:**

We use conventional PCR with 5′-nucleotide tagged primers to generate homologous DNA amplification products from multiple specimens, followed by sequencing through the high-throughput Genome Sequence 20™ DNA Sequencing System (GS20, Roche/454 Life Sciences). Each DNA sequence is subsequently traced back to its individual source through 5′tag-analysis.

**Conclusions:**

We demonstrate that this new approach enables the assignment of virtually all the generated DNA sequences to the correct source once sequencing anomalies are accounted for (miss-assignment rate<0.4%). Therefore, the method enables accurate sequencing and assignment of homologous DNA sequences from multiple sources in single high-throughput GS20 run. We observe a bias in the distribution of the differently tagged primers that is dependent on the 5′ nucleotide of the tag. In particular, primers 5′ labelled with a cytosine are heavily overrepresented among the final sequences, while those 5′ labelled with a thymine are strongly underrepresented. A weaker bias also exists with regards to the distribution of the sequences as sorted by the second nucleotide of the dinucleotide tags. As the results are based on a single GS20 run, the general applicability of the approach requires confirmation. However, our experiments demonstrate that 5′primer tagging is a useful method in which the sequencing power of the GS20 can be applied to PCR-based assays of multiple homologous PCR products. The new approach will be of value to a broad range of research areas, such as those of comparative genomics, complete mitochondrial analyses, population genetics, and phylogenetics.

## Introduction

The arrival of the Genome Sequence 20™ DNA Sequencing System (GS20, Roche/454 Life Sciences) and associated ‘Sequencing-by-Synthesis’ protocol has enabled pyrosequencing of up to 25 million nucleotides in a single four-hour reaction [1]. The method employs single molecule amplification prior to sequencing and therefore eliminates the need for prior cloning. In initial implementations of the technology random fragments from DNA extracts have been sequenced without *a priori* selection of specific genetic regions. As such, all DNA that is present in the sample has a chance of being amplified and sequenced that approximately correspond to its frequency within the DNA extract. The method has proven an efficient tool for use in a number of specific cases, such as the rapid sequencing of relatively small genomes [1], [2].

For purposes such as comparative genomics, mitochondrial sequencing, and population genetics, it is of interest to combine the selectivity of primer-based PCR, with the sequencing power of the GS20 platform. The simplest way to achieve this is the use of the GS20 to emulsion PCR (emPCR) then pyrosequence the products of individual PCR reactions. Due to the sequencing power of the GS20 this approach results in hundreds of thousands of individual sequences from a single PCR reaction, each derived directly from a single original template within the reaction [1]. As such, this result is similar to the generation of sequence data through conventional cloning. We henceforth term the GS20 derived sequences as single molecule sequences. Obviously, in many studies the amount of single molecule sequences produced by single GS20 runs is unnecessary and economically unfeasible, unless several PCR products can be processed simultaneously and correctly assigned.

Thomas and co-authors [3] recently took the first step in making this possible by pooling together eleven PCR products, each targeting different regions of the genome, into single sequencing-by-synthesis reactions using the GS20. In this case, the authors could easily sort the sequence data due to the unique genetic sequence of each original target. Furthermore, by sequencing the combined PCR products from separate individual specimens on specially partitioned fragments (1/8 sections) of the GS20 PicoTitrePlate™, they were rapidly able to generate large numbers of sequences from each of the eleven PCR products (≈1,000 per product) [3].

While this represents an excellent advance in the exploitation of the GS20, in theory the combined “primer specific PCR/GS20” approach can be enhanced even further. For example, the number of sequences generated in even an 1/8^th^ run of the GS20 using a 40×75 PicoTitrePlate™ (currently the smallest commercially available subdivision of a single GS20 reaction) is large; in our experience such a run routinely generates at least 6,000, and more commonly over 10,000 sequences per run. With an estimated 10-fold coverage, using the method of Thomas et al [3] this could enable the pooling of 600 PCR products in a single reaction. However, the subsequent identification of the sequence reads would require either the pooling of 600 PCR products targeting unique genetic regions, or, if multiple homologous PCR products were to be co-sequenced (i.e. multiple different products amplified using a single identical primer pair), an *a prior* knowledge about the exact sequence of each target.

**Figure 1:**
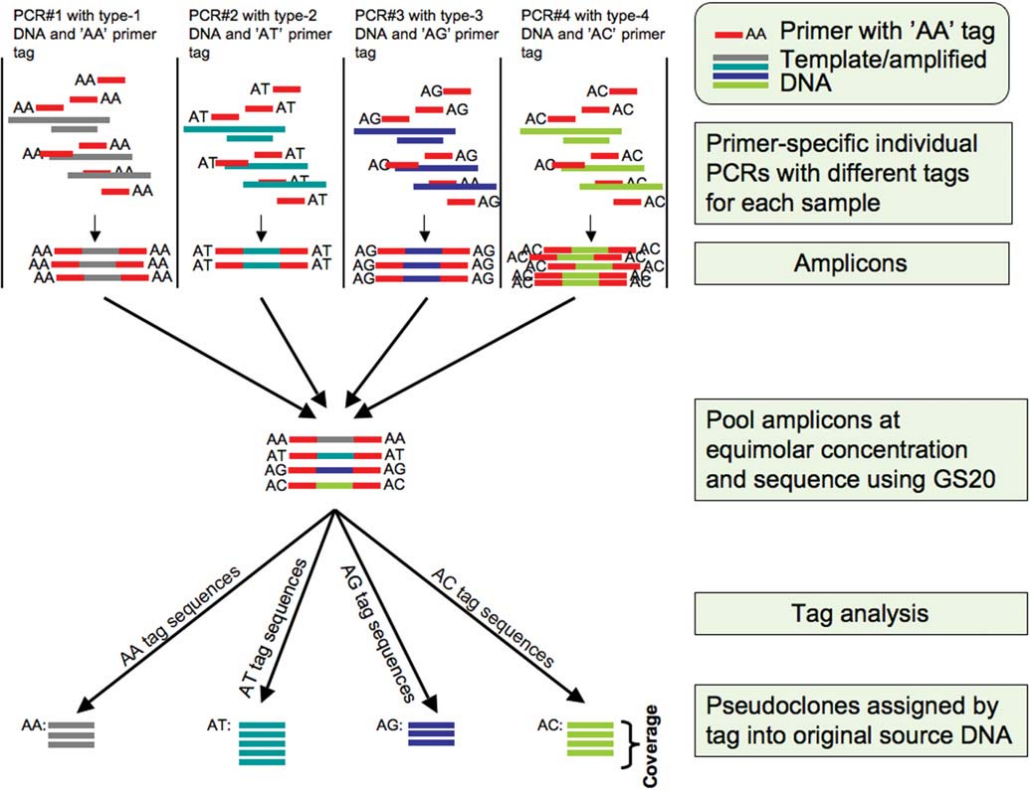
The application of 5′ primer tags to the GS20 sequencing-by-synthesis process.

In this paper we have overcome this problem, presenting a method where initial PCR primers are 5′-tagged with short nucleotide sequences (tags) in such a way that a unique tagged primer combination can be applied to each specific DNA template source. As sequences generated by the GS20 commence at the very first position of the source DNA fragment, the tags are observed in the generated sequences. Therefore sequences can rapidly be sorted into their original template source using the tags (Figure 1). Currently, the method provides a means for the simultaneous sequencing, generation of single molecule sequences, and assignment of short (∼120 bp) from homologous PCR products obtained from multiple individuals. However, as the GS20 sequencing-by-synthesis technologies are developed to increase both the number, and length of the sequences generated, the power of this technique will likewise increase.

## Methods

In theory, a GS20 reaction that has been performed on a pool of different PCR products at equimolar concentration should generate an equal number of sequences from each PCR product. However, in practice it can be expected that random processes occurring during the procedure will result in a Poisson distributed relative frequency of the final products. In addition to this, the different 5′ tags used on the primers for the initial PCR might potentially bias the final sequence distribution. As a result, the incorporation of too many different PCR products in a single GS20 reaction could result in some of them not being sequenced. In contrast, the incorporation of too few PCR products in a single 454 parallel sequencing run minimises the efficiency, and cost efficacy of the method. Furthermore, as one advantage of the approach is the generation of single molecule sequences, it is useful to empirically determine how many sequences can be expected from each of a set of PCR products that are pooled in equimolar concentration.

We performed a test involving the analysis of a single genetic marker in DNA extracts from multiple different individuals to investigate the effectiveness of this method (i.e. how many individual PCR products can be expected to be represented among a set number of sequences). This was achieved using a single conventional pair of mammalian mitochondrial DNA (mtDNA) 16S primers [4]. The primers were originally designed as mammalian generic, and amplify an 89–97 bp fragment (133–141 bp including primers) that is discriminatory between mammalian species. The study is thus an analogue to a likely use of the technique - the PCR amplification and sequencing of specific genetic regions from multiple individuals of a single species.

### 5′ primer tagging

The original primers were modified into sixteen unique forward, and sixteen reverse primers through the addition of 5′ dinucleotide tags (Table 1). In contrast to most conventional sequencing platforms, pyrosequencing methods (such as that used by the GS20) generate data from the first base of the fragment sequenced. Thus, the 5′ tags on each primer will be apparent in the final sequence. The sixteen unique forward and reverse primers can be combined to make 16*16 = 256 unique sequence tags. In this way, an investment of thirty-two initial primers could in theory enable the subsequent discrimination of 256 different products. However, under the current status of the sequencing technology, GS20 sequencing reads are limited to approximately 120 bases, thus in this experiment the full sequence (133–141 bp including primer, species dependent) was not returned and our analyses were limited to simply discriminating using the primer at the sequence end of the product. Furthermore, during the GS20 process, single DNA fragments are mobilised to beads in either orientation (c.f. [1] for details). The implication of this is that approximately 50% of each PCR product will be sequenced from the orientation of the forward primer, and 50% from the orientation of the reverse primer. Hence, this made it necessary to label both the forward and reverse end of each PCR product.

**Table 1:** 5′ tagged PCR primers

Forward primers		Reversed primers	
Name	Sequence (5′–3′)	Name	Sequence (5′–3′)
16Faa	aacggttggggtgacctcgga	16Raa	aagctgttatccctagggtaact
16Fac	accggttggggtgacctcgga	16Rac	acgctgttatccctagggtaact
16Fag	agcggttggggtgacctcgga	16Rag	aggctgttatccctagggtaact
16Fat	atcggttggggtgacctcgga	16Rat	atgctgttatccctagggtaact
16Fca	cacggttggggtgacctcgga	16Rca	cagctgttatccctagggtaact
16Fcc	cccggttggggtgacctcgga	16Rcc	ccgctgttatccctagggtaact
16Fcg	cgcggttggggtgacctcgga	16Rcg	cggctgttatccctagggtaact
16Fct	ctcggttggggtgacctcgga	16Rct	ctgctgttatccctagggtaact
16Fga	gacggttggggtgacctcgga	16Rga	gagctgttatccctagggtaact
16Fgc	gccggttggggtgacctcgga	16Rgc	gcgctgttatccctagggtaact
16Fgg	ggcggttggggtgacctcgga	16Rgg	gggctgttatccctagggtaact
16Fgt	gtcggttggggtgacctcgga	16Rgt	gtgctgttatccctagggtaact
16Fta	tacggttggggtgacctcgga	16Rta	tagctgttatccctagggtaact
16Ftc	tccggttggggtgacctcgga	16Rtc	tcgctgttatccctagggtaact
16Ftg	tgcggttggggtgacctcgga	16Rtg	tggctgttatccctagggtaact
16Ftt	ttcggttggggtgacctcgga	16Rtt	ttgctgttatccctagggtaact
16SF4a	gctacggttggggtgacctcgga	16SR4a	gtacgctgttatccctagggtaact
16SF4b	tcagcggttggggtgacctcgga	16SR4b	tgacgctgttatccctagggtaact
16SF4c	ctagcggttggggtgacctcgga	16SR4c	tagcgctgttatccctagggtaact

In addition to the above experiments, three further unique primer pairs were designed and used for PCR, that contain tetranucleotide tails (Table 1) in order to investigate whether an increased tail length affects the efficiency of the method. Increasing the tag sequence would exponentially increase the number of possible unique primer combinations and thus PCR reactions that can be incorporated into a single GS20 sequencing run.

### DNA samples analysed

DNA from thirteen species was used as PCR template (Table 2). The target species and size of the PCR insert (excluding primers) were as follows: impala (*Aepyceros melampus*) 92 bp; grey wolf (*Canis lupus*) 91 bp; cheetah (*Acinonyx jubatus*) 91 bp; hippopotamus (*Hippopotamus amphibious*) 91 bp; lion (*Panthera leo*) 95 bp; saiga antelope (*Saiga tartarica*) 93 bp, Mueller's Bornean gibbon (*Hylobates muelleri*) 94 bp, narwhal (*Monodon monoceros*) 90 bp; domestic mouse (*Mus domesticus*) 97 bp; musk ox (*Ovibos moschatus*) 93 bp; human 94 bp; Burchell's zebra (*Equus burchelli*) 89 bp; and African buffalo (*Syncerus caffer*) 94 bp. The DNA was extracted from frozen specimens using the DNEasy tissue extraction kit (Qiagen) following the manufacturer's protocol. To increase the number of different PCR products that we could pool into the GS20-reaction beyond a single product from each of available thirteen extractions, we used individual primer pairs on several different extractions each (Table 2).

**Table 2:** Assigned sequence distribution

	Wolf	Cheetah	Hippopotamus	Lion	Saiga	Gibbon	Narwhal	Domestic Mouse	Musk Ox	Human	Zebra	African Buffalo	Impala				
Primer	*Canis lupus*	*Acinonyx jubatus*	*Hippopotamus amphibius*	*Panthera leo*	*Saiga tartarica*	*Hylobates muelleri*	*Monodon monoceros*	*Mus domesticus*	*Ovibos moschatus*	*Homo sapiens*	*Equus burchelli*	*Syncerus caffer*	*Aepyceros melampus*	Total	Correctly assigned	Incorrectly assigned	Assignment Error
16FAA	49	69	23							*1*				142	141	1	0.0071
16RAA	41	58	16											115	115	0	0.0000
16FAC	58	98	23											179	179	0	0.0000
16RAC	21	72	20									*1*		114	113	1	0.0088
16FAG	15	17	36											68	68	0	0.0000
16RAG	20	17	28											65	65	0	0.0000
16FAT	28	44	23				*1*							96	95	1	0.0105
16RAT	18	56	19											93	93	0	0.0000
16FTA				13	64	49					1			127	127	0	0.0000
16RTA				7	39	40	*1*				0			87	86	1	0.0116
16FTC				28	47	19				*1*	19			114	113	1	0.0088
16RTC				32	58	7				*1*	14			112	111	1	0.0090
16FTG				12	57	31					5			105	105	0	0.0000
16RTG				19	55	12					1			87	87	0	0.0000
16FTT				15	54	35					6			110	110	0	0.0000
16RTT				21	48	27					4	*1*		101	100	1	0.0100
16FGA					*1*		86	42	43	19				191	190	1	0.0053
16RGA					*1*		65	54	34	9				163	162	1	0.0062
16FGC							8	64	42	4				118	118	0	0.0000
16RGC							5	63	25	11				104	104	0	0.0000
16FGG							84	51	31	25				191	191	0	0.0000
16RGG							61	61	24	26				172	172	0	0.0000
16FGT							90	43	45	24				202	202	0	0.0000
16RGT							71	46	35	9				161	161	0	0.0000
16FCA						*1*					71	86	80	238	237	1	0.0042
16RCA		*1*									54	96	81	232	231	1	0.0043
16FCC											106	93	106	305	305	0	0.0000
16RCC										*2*	117	108	101	328	326	2	0.0061
16FCG								*1*			80	99	112	292	291	1	0.0034
16RCG											96	82	108	286	286	0	0.0000
16FCT										*3*	86	93	84	266	263	3	0.0114
16RCT										*1*	82	102	74	259	258	1	0.0039
16SF4A											43	2		45	45	0	0.0000
16SR4A											55	5		60	60	0	0.0000
16SF4B	29									*2*	15	4		50	48	2	0.0417
16SR4B	25										19	6		50	50	0	0.0000
16SF4C	51										60	3		114	114	0	0.0000
16SR4C	43										54	3		100	100	0	0.0000
													Total	5642	5622	20	0.1525
													Mean	148.5	147.9474	0.5263	0.0040
													Percent GS20 sequences	83.4	83.1		
																Overall miss-assignment rate	0.003557453
Analysis by Column:														SUM	MEAN		
Correctly Assigned	398	431	188	147	422	220	470	424	279	127	988	782	746	5622	432.46		
Incorrect assigned	0	1	0	0	2	1	2	1	0	11	0	2	0	20	1.5385		
Species assignment error	0.0000	0.0023	0.0000	0.0000	0.0047	0.0045	0.0043	0.0024	0.0000	0.0866	0.0000	0.0026	0.0000		0.0083		

Italic numbers indicate miss-assigned sequences.

### PCR conditions

We generated 64 differently labelled 16S mtDNA PCR fragments (Table 2). PCRs were performed in 25 µl PCR reactions containing 1× PCR Buffer, 2.5 mM MgCl_2_ solution, 0.2 mM dNTP Mix, 1 U Taq DNA Polymerase, 1 µM each primer and 1 µl DNA extract. Cycling was performed using a Mastercycler Gradient Thermal Cycler (Eppendorf) with the following cycle program: Initial denaturation at 94°C for 2 minutes followed by 25 cycles of 94°C for 30 seconds, 56°C for 30 seconds and 72°C for 30 seconds, followed by a final extension of 8 minutes at 72°C. Five µl of the PCR products were visualised on 2% agarose gels using ethidium bromide staining and UV light transillumination. Positive PCR products were purified using the Invisorb Spin PCRapid kit (Invitek) and quantified using a Nanodrop ND-1000 (Nanodrop Technologies). Quantification was performed directly on the purified PCR products (that is, without dilution). Several duplicate measurements indicated that intrasample measurement variation was negligible. Purified yields were between 3.8–26.1 ng/µl (Supplementary Table S1). Subsequently the PCR products were pooled together. The PCR products were at equimolar concentrations (26.1 ng each) with two exceptions; amplification products from the buffalo were added at double concentration (52.2 ng), and PCR products generated from the zebra template used twice the number of different tags. The pooled PCR products were subsequently analysed on the GS20 platform using the complete sample preparation and analytical process, as recommended by the manufacturer (Roche). The initial sample concentration was 9.33 ng/µl and 21 ng (23 µl) was used for the reaction. No nebulization was performed and the average concentration of single stranded library was 75 pg/µl. The calculated dilution factor was 5.25 and sequencing was performed as a full titration run without bead enrichment, i.e. the run was performed on a 40×75 plate, divided into 8 sectors (a titration run uses 4 of these sectors with different numbers of DNA molecules per bead i.e. 1,4,16, and 64 respectively.).

### Conventional sequencing of the targets

Although the complete 16S mtDNA sequences for most of the species analysed is available in the public domain, we regenerated the target sequences for the thirteen mammal species used using conventional dye-labelled sequencing (Sequencing reactions and analyses performed on Applied Biosystems platforms by Macrogen, Korea). This was to ensure that subsequent analyses did not mistake natural sequence variation with sequencing errors. The thirteen individual 16S mtDNA sequences are deposited in GenBank under the accession numbers EF152485–EF152497.

### Initial assignment of the sequence data

As the correct association of tags and sequences is crucial to the approach, we followed very conservative criteria post sequencing in regards to acceptance of the sequence data. Initially, we discarded all sequence reads without an exact match to any of the primers used in the studies (Primer Mismatched Sequences). Subsequently, the identity of the remaining sequences were globally aligned to the thirteen reference sequences (Sanger-sequencing generated) using direct and reverse complementation. The global alignment was performed using ClustalW [5] used the following scoring scheme: matches (+5), mismatches (−4), gap penalty (−10), and a gap extension penalty (−10). The latter penalties were not applied to end gaps. For each alignment a percent identity score was calculated to determine the best match in the following way: excluding end gaps, ambiguous states (Ns) in the 454 sequence, and gaps introduced in the reference sequence during alignment the number of mismatches was calculated.

If a sequence differed at more than one nucleotide from the highest scoring alignment, then the sequences were discarded into a separate dataset. We refer to these sequences as Non-Assigned sequences, and the remaining sequences are referred to as Assigned sequences. The per nucleotide error rate estimated from this type of data is 7.5×10^−4^ [6]. With reads of a length of ≈100 bp excluding primers, and primers of length 22 bp, the expected proportion of non-assigned sequences is then 2.7×10^−3^ and the expected proportion of primer mismatched sequences should be 1.6×10^−2^. Any excess of Non-Assigned or Primer Mismatched Sequences above this level is then due to experimental errors other than sequencing errors, such as contamination.

The identity of the Non-Assigned sequences are of some interest as they may provide information regarding these other sources of experimental error. Thus the Non-assigned sequences were subsequently subjected to BLAST [7] analyses against the NCBI GenBank DNA database in order to determine their identity. During this (and other) BLAST analyses performed, when two or more hits with identical E-score were reported, we prioritised any that matched our 13 target sequences over others.

## Results

### GS20 sequences generated

6765 DNA sequences were generated using the GS20 platform (Sequence Data S1). The sequence data is provided in the supplemental information. The sequencing was performed as a titration run with no bead enrichment and different DNA/bead rations, therefore the number of sequences is lower than what is previously reported for PCR products (8,000–12,000, [3]). As such the calculations of the sequencing efficiency in this study provides a conservative estimate of the potential power of the technique.

### Sequence analysis

#### Primer Mismatch Sequences

Due to the stringent screening criteria applied in this study, 458 (6.8%) of the 6765 initial sequences generated from a 1/8^th^ of a plate run on the GS20 were identified as Primer Mismatch Sequences (see above). These grouped as follows: 377 sequences or 5.6% have sequencing errors in the primer sequence, 54 sequences or 0.8% have the primer sequence starting one position off, 3 sequences or 0.04% have the primer sequence starting two positions off, and 24 sequences or 0.4% have the primer sequence starting more than two positions off. As the theoretically expected number of mismatches based on the sequencing error rate is 1.6%, other sources of error (such as damage to the original DNA template, sequencing errors or mtDNA heteroplasmy) may be affecting the results.

The 458 Primer Mismatch Sequences were identified using BLAST, revealing that 395 of the sequences (86.2%) match the reference sequences of the study. This includes 81 sequences where the primers are as expected, but positioned one or more base pair positions off the 5′ end of the sequence. Among these, 80 sequences match DNA sequences from species used in this study (Supplementary Table S2). Of sequences containing errors in the primers 313 of 377 (83.0%) matched species used in this study (Supplementary Table S3).

That so many sequences contained sequencing errors in the primers (n = 377) was surprising, and warranted further investigation. The sequences could be distinguished into four broad categories as follows: Those that failed show any match to the primer sequences in general (n = 2); Those that were exact match to the core primer but lacked the 5′ tag sequence (n = 121); Those that contained at least one mismatch and no indels (insertion/deletions) (n = 53); and those that contained at least one indel (n = 201) (21 of which also contained a mismatch). We subsequently investigated whether the errors may have arisen during the primer synthesis itself, and not during the sequencing-by-synthesis process. This was tested under the assumptions that a) errors arising during the primer synthesis process would be randomly distributed along the primer sequence, and that b) primers containing errors in the 3′ four nucleotides would bind poorly to the template DNA, thus not enable PCR amplification. If this was the case, then although prior to PCR a random distribution of sequence errors should be observed across the primer sequences, post PCR significantly fewer errors should be observed at the 3′ end of the primer. A χ^2^ test on the distribution of the sequencing errors between the five 3′ terminal nucleotides, the next five (middle) nucleotides, and the remaining 5′ nucleotides confirms that there are significantly fewer sequencing errors in the five terminal 3′ nucleotides of the primers (Pearson's χ^2^ test, χ^2^ = 17.506, p = 0.00001). Therefore the data suggests that at least some of the primer-related errors can be explained by errors during the primer synthesis itself. (We note however that this test was only performed on the primers that contained mismatches without indels, due to the difficulty of accurately aligning the primers that contained indels).

#### Assigned Sequences

The remaining 6307 sequences were identified through a global alignment to the 13 reference sequences. Of these, 5642 sequences (89%) diverged by no more than 1bp from one of the reference sequences, and could thus be assigned to one of the taxa analysed in the study (Table 2).

Twenty sequences (0.4%) were miss-assigned to an incorrect identity. Strikingly, more than half of the miss-assigned sequences (n = 11) are of human origin. Based on the omnipresent nature of human DNA in most laboratory settings, this bias is likely due to contamination during extractions and/or PCR setup. Ignoring all human sequences (n = 138), only 9 sequences could be miss-assigned out of a total of 5504 GS20 non-human sequences (0.00163 percent miss-assignment). Based on a GS20 sequencing error rate of 7×10^−4^ [6], the expected number of miss-assignments due to sequencing errors in the dinucleotide tag is 2×5504× (7×10^−4^)≈7.4 mismatches. Thus, the obtained result is are remarkably close to the expected and miss-assignments of non-human sequences can be explained by sequencing errors alone. This result shows that despite the possibility of sequencing (and other) errors, the assignment based on 5′ tagging is remarkably reliable.

#### Non-Assigned Sequences

Of the 6307 sequences that did not contain a primer error, 665 sequences diverge from the reference sequences by more than 1 bp. However, the expected number of such sequences based on the known sequencing error rate is only 6307×(2.7×10^−3^)≈17, suggesting a significant impact of other factors. Obvious candidates include the amplification of non-targeted genomic sequences (for example laboratory contamination), DNA damage or heteroplasmy in the original template, and errors introduced into the DNA during the initial PCR stage (where a non-proof reading polymerase was used). Of these, 491 sequences (73.8%) match DNA sequences from one of the 13 original taxa amplified by the highest BLAST hit (Supplementary Table S4).

#### Sequence distribution

On average each of the 64 amplicons (grouping forward and reverse reads) had 85× coverage with a standard deviation of 32 (Table 2). The coverage variation is very large. At the extremes we observe that the zebra DNA amplified with a TA tag generating a single forward read and no reverse read, while the zebra amplified with the CC tag resulting in more than 100 forward and reverse reads. There is no evidence that forward or reverse strands are unequally distributed within the sequence dataset (Pearson's χ^2^ test, χ^2^ = 27.2793, df = 18, p = 0.0739).

#### 5′ tag distribution

A Pearson's χ^2^ test strongly rejects an equal distribution among the different tags (χ^2^ = 1725.28, df = 18, p = 0.0). The divergence from the expected numbers are primarily caused by an excess of 5′CN (N representing A,T,G,C) tagged amplicons, and a depletion of 5′TN tags (Table 3), with a small surplus of 5′GN and small depletion of 5′AN tags. We also investigated whether the identity of the second nucleotide within each tag led to non-random distribution of the final sequences. This was achieved using χ^2^ analysis on the 4 independent datasets constituted by the 5′ nucleotide A, C, G and T respectively (i.e. the 4 primer groups AN, CN, GN and TN). The results indicate that with the exception of the 5′ T labelled tags, the sequences were non randomly distributed (AN, χ^2^ = 60.0, d.f. = 3, p = 0.0; CN, χ^2^ = 10.0, d.f. = 3, p = 0.0186; GN, χ^2^ = 16.3, d.f. = 3, p = 0.0009; TN, χ^2^ = 2.35, d.f. = 3, p = 0.5039). Due to the limited number of tetranucleotide tags analysed, we were unable to investigate the effect of the identity of the 3^rd^ and 4^th^ position nucleotides.

**Table 3:** Observed and Expected sequence distributions sorted by 5′ tag composition

5′Tag	Sequences from forward primer	Sequences from reverse primer	Total sequences	Expected sequence frequency	Deviation
AA	141	115	256	274.75	−18.75
AC	179	113	292	274.75	17.25
AG	68	65	133	274.75	−141.75
AT	95	93	188	274.75	−86.75
CA	237	231	468	274.75	193.25
CC	305	326	631	274.75	356.25
CG	291	286	577	274.75	302.25
CT	263	258	521	274.75	246.25
GA	171	153	324	274.75	49.25
GC	114	93	207	274.75	−67.75
GG	166	146	312	274.75	37.25
GT	178	152	330	274.75	55.25
TA	127	86	213	366.33	−153.33
TC	113	111	224	366.33	−142.33
TG	105	87	192	366.33	−174.33
TT	110	100	210	366.33	−156.33
4A*	45 (gcta)	60 (gtca)	105	183,16	−78,16
4B*	48 (tcag)	50 (tgac)	98	274,75	−176,75
4C*	114 (ctag)	100 (tagc)	214	274,75	−60,75
**Total**	**2870**	**2625**	**5495**	**5495**	

* Sequence of the tetranucleotide tag in parentheses

Expected sequence frequencies are calculated to account for the number of initial PCRs commencing from each different 5′tag.

### Effect of 5′ nucleotide on PCR products

In light of the finding that the identity of the dinucleotide tag has an important effect on final sequence distribution, we also performed several statistical tests to investigate whether the 5′ nucleotide of the dinucleotide tag might also affect the initially generated PCR products. Specifically we investigated whether an association exists between the 5′ nucleotide of the tag, and either the final concentrations of the amplified products (ng/µl) or the percent of sequence discrepancies/incorrect templates among the sequences. To test the former, Student's t-tests were performed on the difference in PCR yield (ng/µl) (supplementary data) between primers starting with A, C, G, and T. After Bonferroni correction, only the direct comparison between 5′C and 5′G labeled primers showed any significant difference (A–C p = 0.942, A–G p = 0.397, A–T p = 0.752, C–G 0.003, C–T 0.640, G–T 0.067). To test the latter we investigated whether the number of errors in the sequences are homogeneously distributed among primers starting with A, C, G, and T. A χ2 RxC test indicates that the errors are not distributed homogenously among the primers (χ^2^ = 102.25, p<<0.0001).

To summarise therefore, there is evidence that the identity of the first and second position with the dinucleotide tags affects the final sequence distribution, and the 5′ nucleotide of the tag affects the levels of sequence errors.

### Dinucleotide vs tetranucleotide tag performance

In comparison to the dinucleotide tags, the performance of the tetranucleotide tagged primers was less efficient than predicted (Table 3). Although the small number of tetranucleotide tagged primers tested makes statistically supported comparisons difficult, our observations on the data indicate that overall the rate of sequence miss-assignment for these primers was lower than for the dinucleotide tags.

## Discussion

### Caveats

In this study we present data describing the viability and limitations of a pooled-PCR based approach to GS20 sequencing. Naturally the specific results of this study may be dependent on the genetic region targeted and the PCR primers/target details. As such we caution that while we demonstrate the overall efficiency of this method, future studies are required to confirm the global extent of our observations on the primer efficiencies. In addition, the results of this study are clearly dependent on the quantification method used. In the study we chose the ND-1000 Nanodrop due to its commonplace availability, plus ease and rapidity of use. While more accurate quantification methods such as real-time quantitative PCR may act to reduce the variation between the numbers of single molecule sequenced PCR sequences, the efficiency of using this easily applied technique is in our eyes acceptable, and a great improvement over the costs (both financially and time) that conventional cloning plus sequencing requires.

### Variation in single molecule sequence numbers

Despite pooling the PCR products together in equal concentrations, we find significant variation in the coverage level of the different amplicons, at the extremes varying from a single read to more than 200 reads. This variation is greater than would be expected due to random processes and although there is weak evidence that in some cases the 5′ nucleotide of the tag may play a role, it is more likely an artefact of natural errors. Firstly, some of the variation is likely a result of the ND-1000 measurement accuracy, whether due to the fact that the measurement accuracy of the spectrophotometer (≈±1 ng) is vastly higher than the mass of a single PCR product, or due to the fact that original genomic DNA within the final PCR product lead to overestimation of the amplicon content. Some evidence for this second hypothesis is offered by the finding that although twice as much buffalo DNA was added to the GS20 process, this did not generate twice the number of sequences. Further support for this hypothesis is the observation of different DNA sources among the GS20-sequences (see Supplemental Tables S2, S3 andS4) and human DNA in 7 non-human PCRs. As the majority of the sequences derive from in-study amplicons the carry over of any residual template DNA is unlikely to be a problem with regards to replacing PCR amplicons during the GS20 process. A more likely explanation for the single molecule sequence variations is the effect of 5′-tag composition.

### 5′-tag efficiency

Although we observe all of the originally used 5′-tagged PCR primers among our data, there is clear evidence that some of the tag sequences appear preferable over others during the DNA manipulation processes that occur prior to GS20 sequencing. Most obviously, we note that dinucleotide tags containing the 5′-CN motif are significantly overrepresented in the final data. In contrast, those with the 5′-TN motif appear significantly underrepresented. Furthermore, there is evidence that the second base in the tag is also plays an affect on the sequencing efficiency. As mentioned previously, we caution however that this analysis is based on a single primer set and on one GS20 analysis, and as such, may be specific to this analysis. However, we speculate that a potential cause of the bias may be due to the GS20 DNA preparation processes. During the first stages of DNA preparation for the GS20 run, DNA is blunt ligated to adaptor sequences using the T4 Ligase enzyme Quick Ligase (New England Biolabs), followed by 3′ nick repair using *Bst* DNA polymerase, Large Fragment (New England Biolabs). Following this process all DNA is ligated to similar ligator sequences, thus this is most likely the stage where the selection occurs. As such, it would seem that the ligase has some propensity for 5′ C-labelled DNA. Under this hypothesis, it would seem logical that a similar bias should exist in DNA sequences generated from unamplified DNA using the GS20. This does not seem to be the case however. In an examination of the frequency distribution of the 5′ base of a dataset of 1214 recently published GS20 generated mammoth mtDNA sequences [6]) we find no significant difference between the observed and expected base frequencies (Calculated factoring in the total sequence base composition, Chi Squared Test p = 0.01). As such, in the absence of further studies to confirm the pattern we remain unable to explain our observations.

### Sequence Errors

Overall we found that 89% of the total sequences generated in this dataset were identical or very close (1 bp divergence) from the target sequences. This finding was based upon our conservative data assignment criteria, and without the use of a PCR enzyme with proof-reading capacity for the initial PCRs (the use of which is likely to increase the sequence accuracy). Furthermore, in an applied experimental situation the single molecule sequences produced from each PCR product could be used to further aid the assignation of target sequences. From a practical point of view, this error rate will have ramifications on study design, with regards to the numbers of single molecule sequences required per individual. This will be exceedingly important in situations where variation is expected in the target PCR products, for example studies on allelic variation (whether due to natural or artificial genetic variation).

The 11% of the sequences that we allocated to the primer mismatch or non-assigned data sets were broadly comprised of 3 groups of sequence. Of these, two (rejections due to primer or amplicon sequencing errors) are likely artefacts of the current GS20 pyrosequencing chemistry, or due to errors in the original primer synthesis process. The third group are sequences that did not BLAST against our 13 target species as the closest hit. This might be explained due to several reasons. First is contamination arising during the extraction or PCR setup. This is clearly evident in the sporadic human DNA presence, although also supported in BLAST results that clearly match other species, for example the occurrence of waterbuck (*Kobus ellipsiprymnus*) sequences (a species that has been worked on previously in our laboratories). Clearly these sequences could be minimised through the adoption of stricter PCR setup protocols. An additional cause for the results may also be the fact that many species are closely related in the targeted 16S mtDNA region, and as such small modifications in the sequences due to DNA damage, heteroplasmy, or PCR enzyme error might lead to erroneous sequence identification.

### Applied use of the technique and future prospects

In response to our observations on how the specific tag sequence affects sequence errors and final sequence distribution, and as to the apparent reduced miss-assignment rates of the tetranucleotide tags, we suggest the following primer design guidelines. In the absence of specific information as to the behaviour or individual tags, it is difficult to pool the products at specific predetermined ratios in order to account for the sequencing behaviours. However, as there appears to be a strong effect that the final 5′ nucleotide has on the sequencing efficiency, we suggest that the ultimate 5′ nucleotide should be conserved among the different tag to give a more uniform distribution of the GS20 sequences. Although C provides the highest levels of sequences in our data, our data indicates that 5′-TN tags show little or no effect of the second nucleotide on final sequence distribution, thus may ultimately prove more useful. With regards to tag length, our limited observations are that increased length reduces the competitive binding efficiency of the PCR product (against shorter tags) indicates that all PCR products should incorporate a tag of identical length (the length will depend on the number of different tags required). The tetranucleotide tags performed well in the initial specific PCRs, yielding 7–26 ng/µl purified DNA. With a fixed 5′ nucleotide, these would still enable 4̂4 (64) different forward and reverse primers to be used, which in combination enable the generation of 64*64 = 4069 identifiable PCR products (assuming that the reverse primer sequence is returned by the GS20). If tagged PCR products targeting multiple different genetic regions are also pooled (either generated through single- or multiplex PCR assays), then clearly the number of products in a single reaction increases dramatically.

### Conclusion

In this study we demonstrate the principal of the application of 5′ tagged PCR primers in the sequencing of homologous PCR products on the GS20 platform. As we have noted several times, our observations on the method kinetics are preliminary, and more detailed follow up studies will be required to clarify the power of the method. Furthermore, as improvements are made in sequencing-by-synthesis methods, the efficiency and power of the 5′tagged PCR method is predicted to increase. Based on our data however, it is possible to provide a more quantitative assessment of the efficacy of the method as a sequencing platform. A single run of the GS20 platform can generate 25 million nucleotides [1]. Taking into account an 89% efficacy, the average alignment depth for 50 mitochondrial chromosomes each of 16 kbp length, will be 27.8×. Based on our distribution of number of sequences obtained from each species, we would expect the specimen with the most shallow coverage among the 50 specimens to have a coverage of 11×. If the mitochondrial genome has been sequenced in units of 100 bs, the chance that any part of the chromosome has not a coverage of at least 1 is then 1−(1−e^−11^)^160^ = 2.7×10^−3^. These numbers may improve as the experimental techniques also improve, for example by pooling DNA considering the dinucleotide motif.

At the moment, it is clear that the GS20 platform should be preferable in most studies where cloning otherwise would be required. Our results also suggest that even when taking into account problems relating to unequal pools of DNA or differences due to dinucleotide motif, the GS20 platform provides a time effective alternative to Sanger sequencing. As the pricing of the method decreases, it may also become more cost effective than Sanger sequencing. In conclusion, we believe that this new approach combining 5′tagged PCR with GS20 sequencing will be of importance to a broad range of research areas where large-scale comparisons of homologous DNA sequences from multiple sources are needed such as is the case in comparative genomics, population genetics, and phylogenetics.

## Supporting Information

Table S1 DNA concentrations of original PCR products
(0.04 MB XLS)

Table S2 Blast Results for the 81 sequences that have an exact primer match but are >1 nucleotide offset from 5′ end of the sequence.
(0.05 MB XLS)

Table S3 Blast Results for the 377 sequences that did not have an exact primer match.
(0.12 MB XLS)

Table S4 Blast Results for the 665 sequences that had an exact primer match but were excluded because of more than 1 mismatch with reference sequences.
(0.17 MB XLS)

Sequence Data S1 Fasta file
(1.18 MB RTF)
